# An improved method of anchoring chest drain and suture technique for Uni-portal VATS incision

**DOI:** 10.1007/s11748-021-01699-x

**Published:** 2021-09-13

**Authors:** Zhiyong Sun, Jiajie Zheng, Ziang Cao, Xiaojing Zhao

**Affiliations:** Department of Thoracic Surgery, Renji Hospital, Shanghai Jiao Tong University School of Medicine, No. 160 Pujian Road, Shanghai, 200127 China

**Keywords:** Uni-portal, Suture, Chest drain

## Abstract

Uni-portal video-assisted thoracoscopic approach is currently a popular surgical technique in general thoracic surgery. After operation, a chest tube is usually placed through the incision to drain the effusion and gas from the thoracic cavity. In the conventional method, the retaining stitches should be taken out ten days after removing chest drain. To get better would-healing and avoid unsightly scar, we explored a method of anchoring chest drain and two-layer suture for Uni-portal incision without removing stitches post operation.

## Introduction

Uni-portal video-assisted thoracoscopic surgery has been widely carried out all over the world. Compared with multi-portal video-assisted thoracoscopic approach, a single surgical incision can avoid multi-level intercostal nerve injury and improve the esthetic satisfaction of patients.

In a conventional method, the chest tube is placed through the surgical incision, and fixed on both sides of the tube. This method of fixation is easy for junior surgeons to master. When extubating, the patient need to take a deep breath. However, if the doctor is unskilled or the patient does not cooperate well, the risk of pneumothorax and effusion leakage will increase. Ten days after removing the chest drain, the retaining stitch should be taken out.

**Fig. 1:**
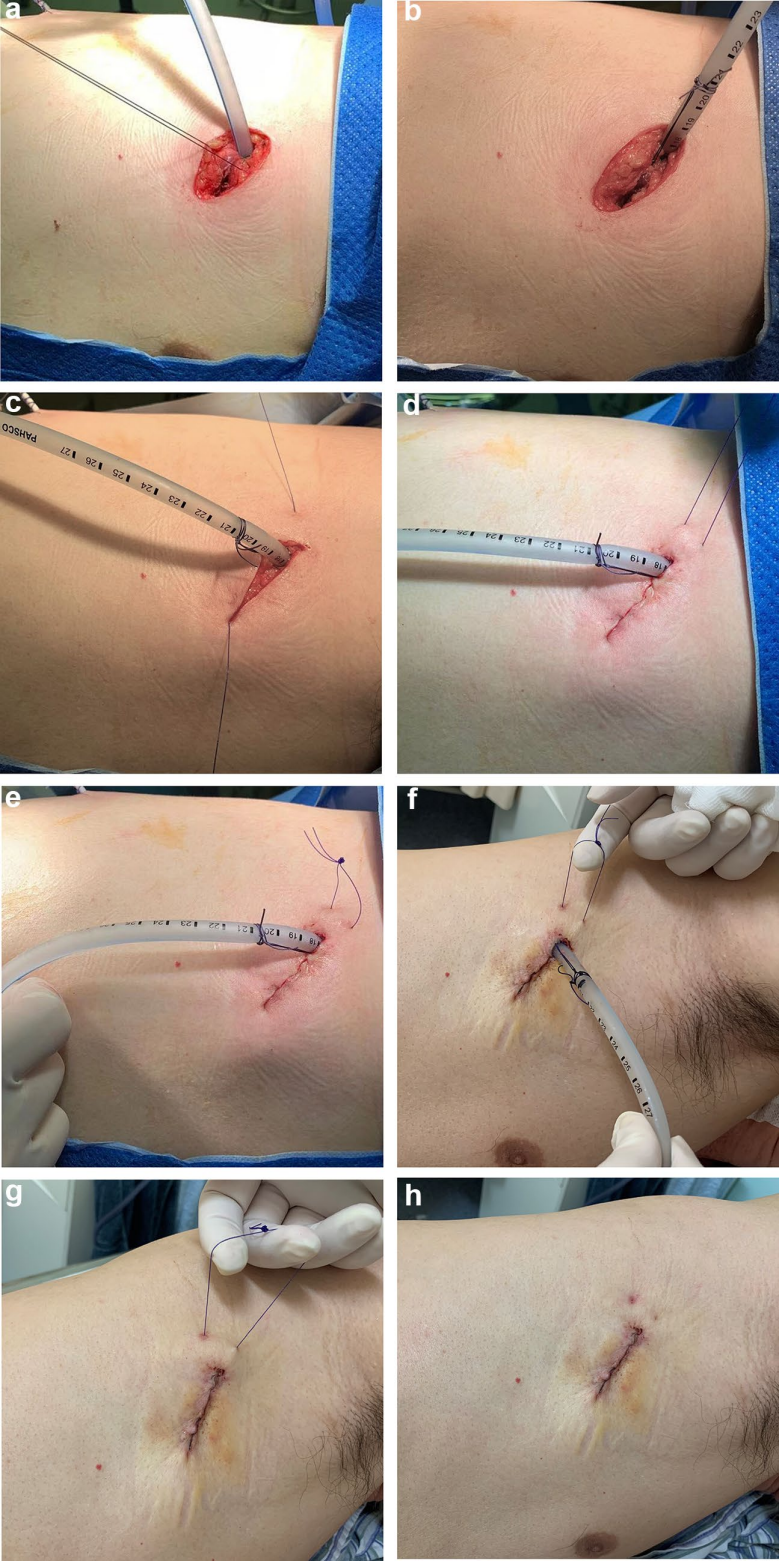
**a** Suture the muscles on both sides of the incision with an 1–0 absorbable suture (VicrylTM Plus,ETHICON); **b** Suture was tied to anchor the chest tube as a fixed line; **c** Continuously suture the subcutaneous tissue from the front side to the backside; **d** Close the skin and take out the needle from the backside; **e** Tied the two sutures and removed the needles; **f** Cut the fixed suture and pull up the suture buckle with your finger; **g** Tighten the barbed suture under the skin and subcutaneous tissue; **h** Cut the sutures outside of the skin

We summarized an improved method, anchoring chest drain and two-layer suture technique, does not need the stitch out for Uni-portal video-assisted thoracoscopic incision (Fig. 1).

## Technique

First, we sutured the muscles on both sides 1 cm from the posterior edge of the incision with an 1–0 absorbable suture (VicrylTM Plus, ETHICON). Vicryl Coated VICRYL™ elicits a minimal acute inflammatory reaction in tissue and ingrowth of fibrous connective tissue. Then, a 20–24 Fr chest tube was placed through the incision. After adjusting the position of the chest drain, the previous suture was tied to anchor the chest tube. Similarly, another 1–0 absorbable suture (VicrylTM Plus, ETHICON) was used to continuously close the muscle tissue.

**Fig. 2:**
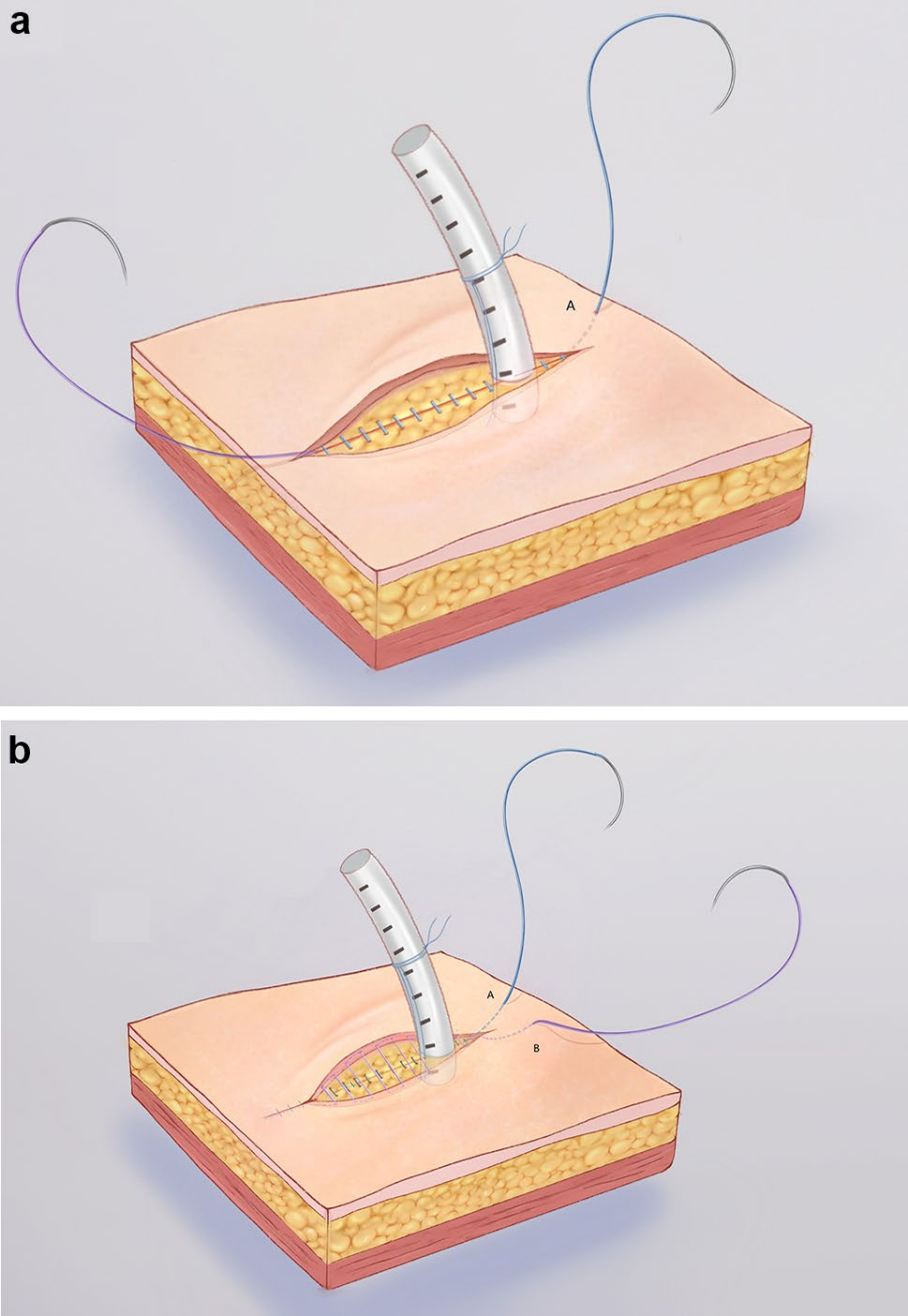
**a** Continuously close the subcutaneous tissue (blue part); **b** Horizontally close the skin (purple part)

The subcutaneous tissue and skin were sutured with a 3–0 barbed absorbable bidirectional device (Stratafix Spiral PDO, ETHICON) (Fig. 2). One barbed suture needle was inserted subcutaneously from the front side of the incision. Pulling the suture until the barb locks, and continuously closing the subcutaneous tissue to the backside (Point A) 1 cm from the posterior edge of the incision. Then we used the other half of the barbed suture in the same way to horizontally close the skin and take out the needle from the backside (Point B). Tied the two sutures and removed the needles. After completely closing the incision, a buckle can be seen at the end of the incision. When suturing the skin near the chest drain, pay attention to fully pulling the skin with tweezers so as to obtain a good vision and avoid suturing the chest drain.

When pulling out the chest tube, cut the fixed suture first. After removing the drain, pull up the buckle with your index finger, which can tighten the barbed suture under the skin and subcutaneous tissue at the same time. Finally, cut the sutures on the outside of the skin.

We use a barbed bidirectional suture to close both layers at the same time, which is different from the others [1] who close the skin tissue only with the barbed suture. When the suture buckle is pulled up after extubation, two layers can be tightened at the same time, so as to strengthen the alignment of tissues and reduce the risks of incision complications. With our technique, there is no need to take out stitches after discharge.

## Comment

The concept of Uni-portal video-assisted thoracoscopic surgery was first proposed by Dr. Gaetano Rocco [2] more than ten years ago, and Diego Gonzalez-Rivas [^3^] in Spain has been promoting the procedure for years. Nowadays, Uni-portal video-assisted thoracoscopic surgery has been accepted and applied by more and more thoracic surgeons due to its less-invasiveness.

Chest drain is required after routine thoracic surgery. The main purpose of chest drain is to evacuate air or fluid and monitor the condition of intrathoracic space. It is really important to secure the tube tightly and remove it without any complications [4]. The conventional fixation methods are different from each center. The British Thoracic Society guidelines suggested that the vertical mattress suture is employed and it is appropriate for a linear incision while they remove the chest tube [5]. In our center, when extubating, the doctor should instruct the patient to inhale deeply, pull out the tube quickly and bandage the wound. After the incision heals, remove stitches.

We found that in some cases who were fixed in the conventional method, the skin around the tube is not well aligned. Short-term cutting of skin with fixation suture of the tube will lead to poor incision healing after stitches removal, and improper work also will lead to gas and effusion leakage. Although this phenomenon is not as common as subcutaneous emphysema after extubation, it still requires our attention.

There are many papers focusing on improved suture methods, among which we refer to the suture technique of Zhong Wenzhao's team [6] and make our improvement. Unlike Zhong’s method, we chose the barbed absorbable bidirectional suture (Stratafix Spiral PDO, ETHICON). The middle point of this suture is defined by barbs facing in two different directions, which is different from the previous single-direction barbed line. Taking advantage of this feature, we succeed in finishing two-layer suture by this device. The unique barbed structure is helpful for the healing of incision after extubation, and the bidirectional structure can suture the subcutaneous tissue and skin layer at the same time. Our technique simplifies the process of removing chest drain. Different from the conventional method of anchoring chest drain, patients do not need to inhale deeply and hold their breath during extubating. More importantly, after pulling out the tube, there is no need to knot and suture. This will make it easier and safer for junior residents to remove the catheter and avoid complications associated with the removing procedure. Because of this special suture method, especially the narrow space near the chest drain, the surgeon needs to spend an extra 5–10 min to close this incision. According to the experience of our center, through the suture training of 3–5 cases, junior residents could also master this technique.

The alignment of the two layers is strengthened, and the risks of incision complications are reduced. This suture and fixation method also avoids removing stitches on follow-up, which makes the incision heal better and improves the esthetic satisfaction of patients.
